# Survival Benefit and Safety of No. 10 Lymphadenectomy for Gastric Cancer Patients With Total Gastrectomy

**DOI:** 10.1097/MD.0000000000000158

**Published:** 2014-11-28

**Authors:** Kun Yang, Wei-Han Zhang, Xin-Zu Chen, Xiao-Long Chen, Bo Zhang, Zhi-Xin Chen, Zong-Guang Zhou, Jian-Kun Hu

**Affiliations:** From the Department of Gastrointestinal Surgery, West China Hospital, Sichuan University, Chengdu, Sichuan Province, China (KY, W-HZ, X-ZC, BZ, Z-XC, Z-GZ, J-KH).

## Abstract

This study was aimed to evaluate the survival benefit and safety of No. 10 lymphadenectomy for gastric cancer patients with total gastrectomy.

Splenic hilar lymph nodes (LNs) are required to be dissected in total gastrectomy with D2 lymphadenectomy. However, there has still not been a consensus in aspects of survival and safety on No. 10 LN resection.

From January 2006 to December 2011, 453 patients undergoing total gastrectomy for gastric cancer were retrospectively analyzed. Patients were grouped according to No. 10 lymphadenectomy (10D+/10D−). Clinicopathologic characteristics were compared between the 2 groups. These patients had undergone a follow-up until January 2014. The overall survival, morbidity, and mortality rate were analyzed. Subgroup analyses which were stratified by the sex, age, tumor location, lymphadenectomy extent, curative degree, differentiation, tumor size, and TNM staging (ie, stages of tumor) were performed.

There were 220 patients in 10D+ group, whereas 233 in 10D− group. In terms of prognosis, the baseline features between the 2 groups were almost comparable. The incidence of No. 10 LN metastasis was 11.82%. There was no difference in morbidity and mortality between the 2 groups. Significantly more LNs were harvested from patients in 10D+ group (*P* = 0.000). The estimated overall 5-year survival rates were 46.44% and 37.43% in 10D+ group and 10D− group respectively, which is not statistically significant (*P* = 0.3288). Although no statistical significance was found in the estimated 5-year survival rate, these data were obviously higher in patients with age >60 years, Siewert II/ III tumors, N1 status, or IIIa/IIIc stages when No. 10 lymphadenectomies were performed.

Although the differences were obvious, the 5-year survival rates between the 2 groups did not reach statistical significances, which was probably caused by too small patient samples. High-quality studies with larger sample sizes are needed before stronger statement can be done. Until then, the No. 10 LNs’ resection might be recommended in total gastrectomy with D2 lymphadenectomy with an acceptable incidence of complications.

## INTRODUCTION

Gastric cancer is a disease with a high incidence. It is estimated that approximately 22,220 new cases of gastric carcinomas and 10,990 deaths would occur in the United States in 2014.^1^ In contrast to a decline in the incidence of gastric carcinoma, there has been a proximal migration of carcinoma in the Western countries.^2–4^ Surgery is the mainstay of treatment for patients with gastric carcinoma. Nowadays, D2 lymphadenectomy is generally accepted as the standard surgery for gastric carcinoma in East Asia. According to the Japanese treatment guideline of gastric cancer,^5^ total gastrectomy and D2 lymphadenectomy should be often adopted for middle third gastric carcinoma, advanced esophageogastric junction tumor, or huge tumors. Therefore, the proportion of total gastrectomy has been increasing in the ensuing years.

Lymph node (LN) metastases are found more frequently in the splenic hilum (No. 10 LN) in the proximal gastric and gastroesophageal junction cancers.^6^ Several studies have been reported stating that the incidence of LN metastasis is 9.8% to 20.9% at the splenic hilum in the advanced proximal and middle third gastric carcinomas.^7–9^ Due to the high frequency of LN metastasis, splenectomy is performed for the purpose of effective LN dissection at the splenic hilum concurrently. However, it has been reported that splenectomy could not show a superiority on survival rates compared with that of splenic preservation, and thus routinely performance of splenectomy should not be recommended.^10,11^ Therefore, spleen-preserved lymphadenectomy is proposed and applied therein.

Although splenic hilar LNs are required to be dissected in D2 lymphadenectomy when total gastrectomy is performed according to the Japanese gastric cancer treatment guideline 2010 (version 3) by the Japanese Gastric Cancer Association (JGCA)^3^; the survival benefit of No. 10 lymphadenectomy is still controversial and the related data or evidence are rare. In addition, the implementation status of No. 10 lymphadenectomy in total gastrectomy with D2 lymphadenectomy is also varied. Some researches have demonstrated that spleen-preserved lymphadenectomy was necessary to achieve radical resection in the advanced middle third gastric carcinoma patients with risk factors.^12^ It has been reported that limited No. 10 LN resection might be accepted for upper and middle third-stage cT1–2 gastric cancer, rather than cT3 disease.^13,14^ Others reported that the prognosis of patients with LN metastasis at the splenic hilum was significantly poorer compared with that of patients with metastases in the other extraperigastric nodes wherein No. 10 LN metastasis should also be considered as one of the incurable factors.^15–17^ Splenic hilar LN dissection may be omitted without decreasing curability in patients with Siewert type II adenocarcinoma of the esophagogastric junction (AEG).^18^ Furthermore, performance of No. 10 lymphadenectomy might indicate more morbidity and mortality because of complicated anatomy at splenic hilum.

Therefore, there still has not been a consensus in aspects of survival and safety on No. 10 LNs’ resection. The aim of this study is to evaluate the survival benefit and safety of No. 10 lymphadenectomy for gastric cancer patients with total gastrectomy.

## METHODS

### Patients

From January 2006 to December 2011, a total of 453 patients with gastric carcinoma who underwent total gastrectomy were retrospectively analyzed. Patients were divided according to No. 10 lymphadenectomy (10D+/10D−). The preoperative diagnosis of gastric carcinoma was confirmed by gastric endoscopy followed by biopsy. Patients diagnosed with other gastric malignances such as lymphoma, gastrointestinal stromal tumor, and any previous malignancy or secondary malignancies other than primary gastric carcinoma were excluded.

### Surgical Techniques

In this study, all patients underwent total gastrectomy with D1, D1,+ or D2 LN dissection for gastric cancer as defined by the Japanese Classification of Gastric Cancer Association.^5^ Roux-en-Y esophagojejunostomy was performed to reconstruct the digestive tract. Pancreatectomy or splenectomy was done only when there was invasion of the pancreas or spleen, or to enable en bloc dissection of obvious metastatic No. 10 LNs. Most patients underwent spleen-preserved lymphadenectomy to dissect the lymphatic tissue at the splenic hilum without sacrificing the spleen and splenic vessels. The grouping rule of regional LNs was according to the Japanese classification of gastric carcinoma (third English version) by JGCA.^19^ All the operations were performed by an expertise of surgeons specialized in gastrointestinal surgery, at the West China Hospital, Sichuan University, Chengdu, China.

### Follow-up

Patients underwent a follow-up, which was done by telephone calls, letters, or outpatient visits. As of January 2014, the overall follow-up rate was 88.1% (399/453) and 11.9% (54/453) of patients were lost to follow-up.

### Clinicopathologic Analysis

The clinicopathological features, such as sex, age, tumor size, tumor location, Borrmann type, depth of tumor invasion, LN metastasis, staging, morbidity, mortality, and survival outcome were collected from the database and compared between 10D+ group and 10D− group. Metastatic ratio of LNs was defined as the ratio of the number of patients with metastatic LNs over the number of patients with No. 10 lymphadenectomy; whereas Metastatic degree of LNs was defined as the ratio of the number of metastatic LNs over the number of harvested LNs. Clinicopathologic terminology was based on the Japanese Classification of Gastric Carcinoma (third English version).^19^

### Statistical Analysis

SPSS 11.5 software (SPSS Inc, Chicago, IL) was used for statistical analyses. Quantitative variables of normality were tested, while confirming the normal distribution, where data are expressed as means ± standard deviation. Two independent *t* tests were performed, or data were expressed as medians with a range taking the Spearman test into consideration. For categorical data, the *χ*^2^ test was used to compare frequencies. Survival was calculated by Kaplan–Meier estimation and the log-rank test. A *P* value of <0.05 (2-sided) was considered statistically significant.

## RESULTS

### Patient Characteristics

There were 220 patients in the 10D+ group and 223 in the 10D− group. The general clinicopathologic characteristics are summarized in Table 1. The proportions of men and women in the 2 groups were similar. Although the tumor locations in the 10D+ and 10D− groups were significantly different (*P* = 0.000), the age, sex, and comorbidity were comparable between the 2 groups. There was no significant difference in the degree of LN resection, curative degree, combined organ resection, differentiation, tumor size, depth of invasion, LN metastasis status, and TNM staging between the 2 groups (Table 1).

**TABLE 1:**
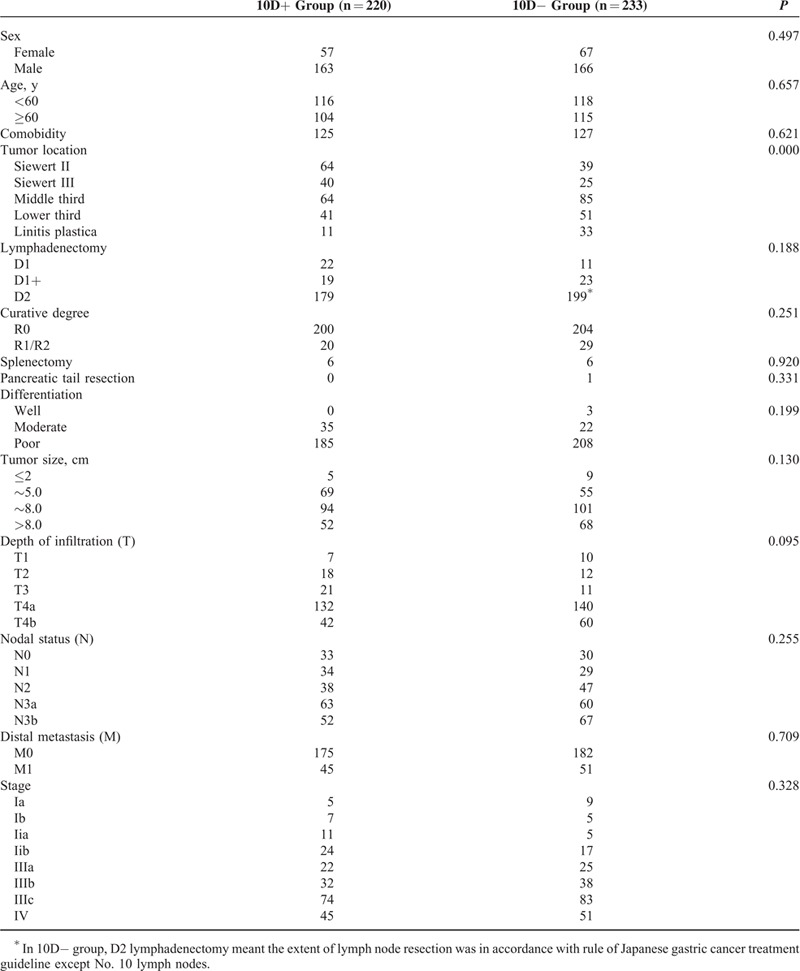
General Clinicopathological Characteristics of the Patients

### Metastatic Ratio and Degree of Lymph nodes

In 220 patients with No. 10 lymphadenectomy, the dissected No. 10 lymph nodes of 106 patients were proved to be fatty tissues by histological examination. There were 26 patients with No. 10 LN-positive metastasis and the metastatic ratio was 11.82% (26/220). A total of 234 LNs at the splenic hilum were harvested with 53 involvements. The metastatic degree was 22.65% (53/234). We could also see in those patients, who had a positive metastasis of No. 10 LN, that the tumor was larger, depth of serosa invasion was greater, the rate of LN involvement was higher, and the advanced stages were more in frequency.

### Operative Variables

The mean number of harvested LNs were 35.09 ± 13.82 in 10D+ group versus 27.01 ± 11.86 in 10D− group (*P* = 0.000). There were no significant differences in the volume of intraoperative blood loss (*P* = 0.087) and operation time (*P* = 0.387) between the 2 groups. The postoperative hospital stays were 10.97 ± 4.06 days and 11. 46 ± 6.12 days in the 10D+ group and 10D− group, respectively (*P* = 0.336). The numbers of patients to be reoperated in the 2 groups were not statistically different (*P* = 0.331). The details can be seen in Table 2.

**TABLE 2:**
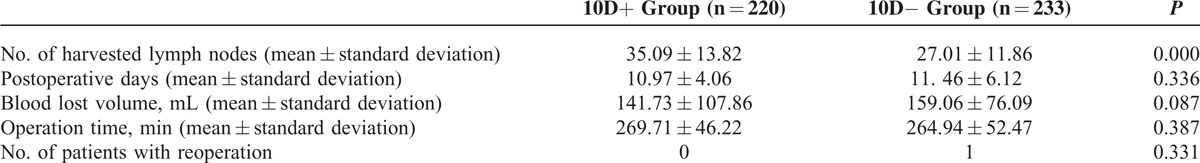
Operative Variables of the Patients

### Morbidity and Mortality

One patient from 10D+ group suffered from splenectomy due to intraoperative splenic injury while dissecting No. 10 LN. And there was no intraoperative splenic injury in 10D− group. The overall postoperative morbidity rates were 14.09% versus 12.02% in the 10D+ and 10D− groups (*P* = 0.512), respectively. Postoperative complications consisted of anastomotic fistula, wound infection, intraperitoneal infection, digestive hemorrhage, biliary fistula, lymphatic fistula, intestinal obstruction, pulmonary infection, hiccup, diarrhea, arrhythmia, delirium, and bacteremia (Table 3). One patient from the 10D+ group died due to postoperative respiratory failure. The postoperative mortality was 0.45% versus 0% in the 10D+ and 10D− groups (*P* = 0.303), respectively.

**TABLE 3:**
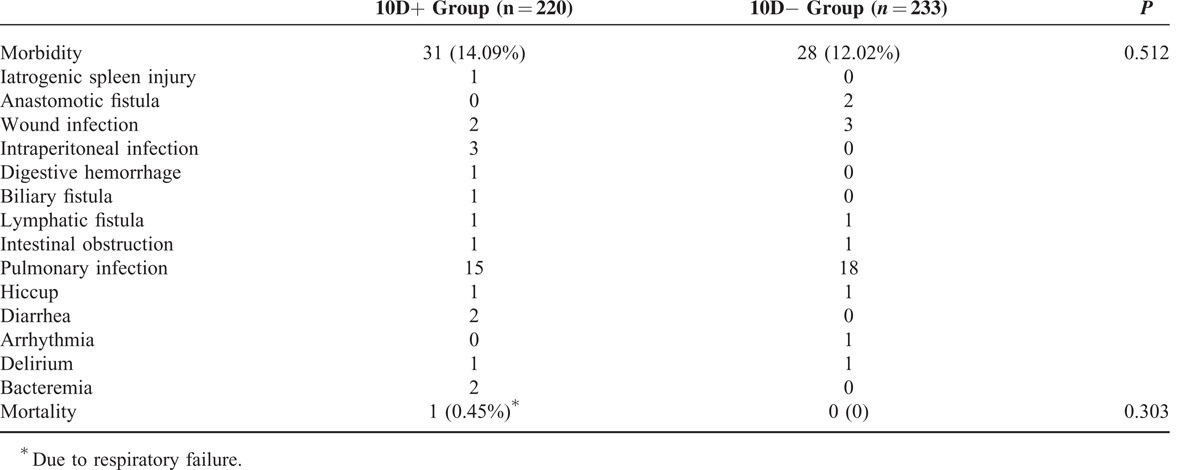
Morbidity and Mortality

### Long-term Survival

**FIGURE 1:**
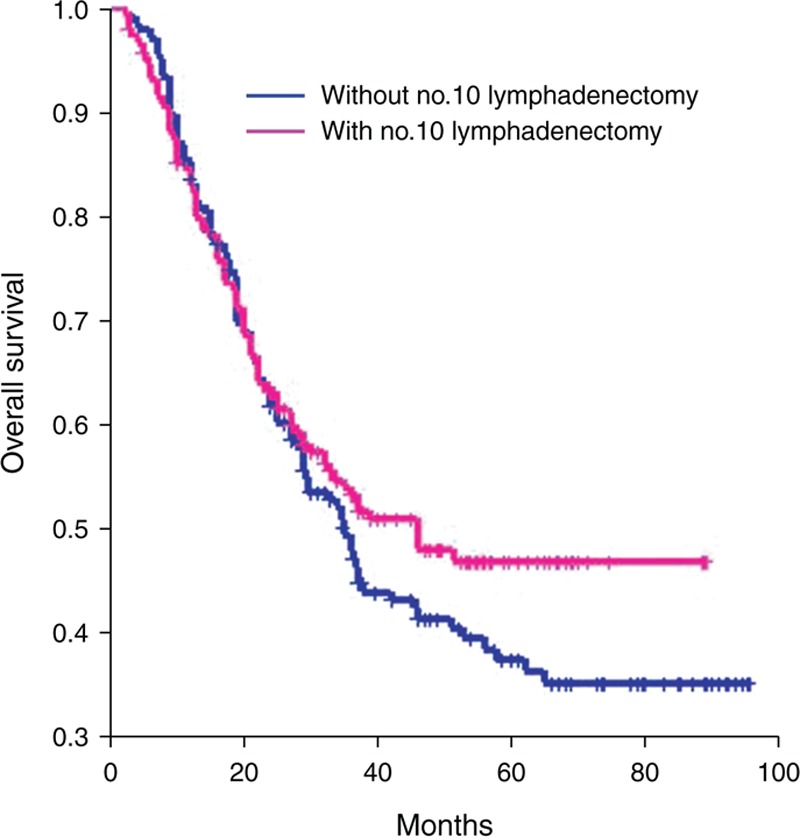
Survival curves of the 2 groups (*P* = 0.3288).

As of January 2014, 104 patients from the 10D+ group and 113 from the 10D− group had died. The estimated 5-year survival rates for patients with and without No. 10 lymph nodes resection were 46.44% and 37.43%, respectively. The overall 5-year survival rate was slightly better in the 10D+ group, but this was not statistically significant (*P* = 0.3288) (Figure 1).

Stratified by clinicopathologic factors, there were no significant differences in the estimated 5-year survival rates between the 2 groups. The results of subgroup analyses are summarized in Table 4. Although not statistically significant, the estimated 5-year survival rates were often higher, especially in patients with ages >60 years, Siewert II/ III tumors, N1 status, or IIIa/IIIc stages, when No. 10 lymphadenectomies were performed. When considering some poor prognostic factors, such as R1/R2 resection, T4b, N3b, M1, or IV stage, the 5-year survival rates of patients in 10D+ group were even lower than those of 10D− group without any significance.

**TABLE 4:**
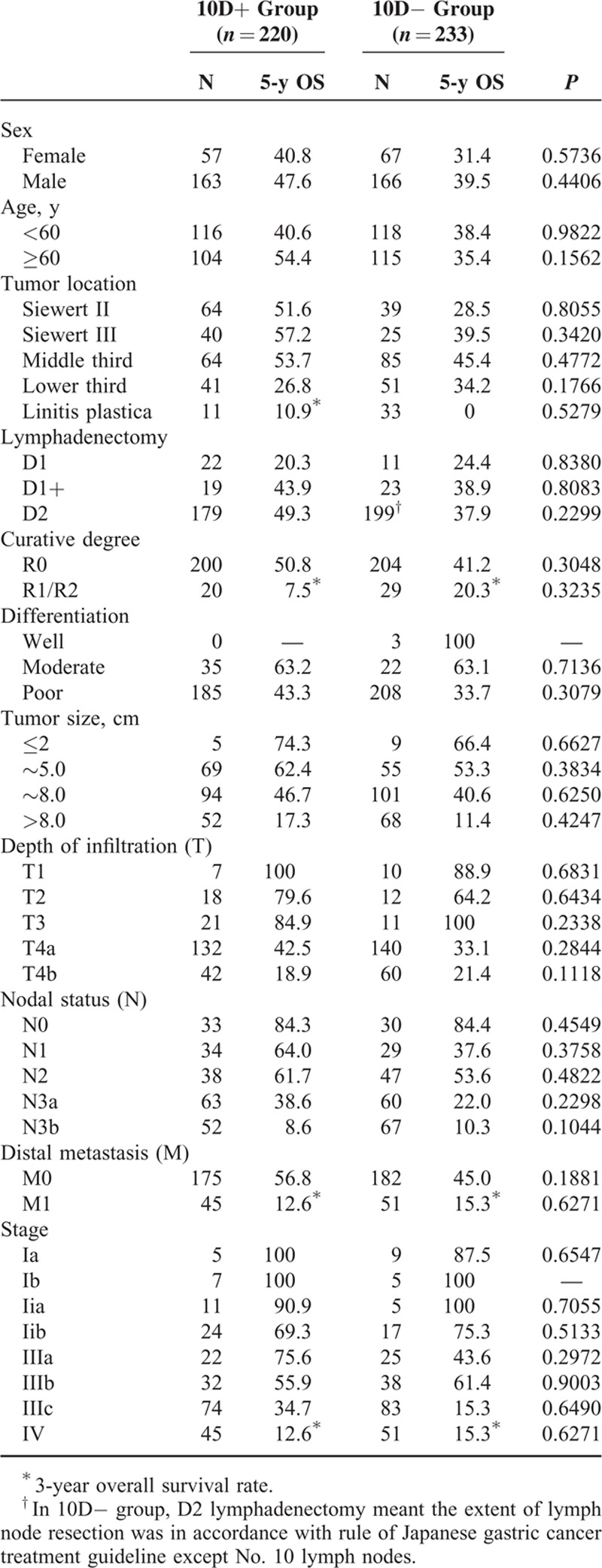
Survival Analysis Stratified by Clinicopathologic Factors

## DISCUSSION

The incidence of proximal gastric cancers has increased.^2–4^ Now, the incidence of LN metastasis has been reported to be 9.8% to 20.9% at the splenic hilum.^7–9^ Due to the high frequency of LN metastasis at the hilum, No. 10 lymphadenectomy is an important surgical consideration for patients with advanced middle third gastric cancer.^20^ As for a curative total gastrectomy, it is necessary to dissect the LNs in the splenic hilum according to the Japanese gastric cancer treatment guideline 2010 (version 3).^5^ Splenectomy has been recommended to facilitate No. 10 LNs’ dissection from a long time duration. However, many reports have showed no benefits from routine or prophylactic splenectomy versus spleen preservation.^10,11^ So, spleen-preserved lymphadenectomy has been prosposed. However, from another aspect, it has been reported that the prognosis of patients with LN metastasis at the splenic hilum was significantly poorer compared with that of patients with metastases in the other extraperigastric nodes and splenic hilar LN metastasis, which should be considered as one of the incurable factors.^15–17^ Also, some researches have proved that splenic hilar LN dissection may be omitted without decreasing curability in patients with Siewert type II AEG.^18^ Therefore whether No. 10 lymphadenectomy could bring survival benefit for gastric cancer patients with total gastrectomy is still controversial.

In this research, our results have showed that the incidence of No. 10 LN metastasis was 11.82%, which was in accordance with other researches.^8,9,20^ And the metastatic degree was 22.65%, which indicated that the dissection of No. 10 LNs should be given a heed to.

Nowadays, most of the research focuses on the comparison of overall survival rates in patients with or without No. 10 LN involvement, or uses the index of estimated benefit from LN dissection to assess the efficacy of No. 10 LN dissection. Studies investigating the impact of No. 10 lymphadenectomy on survival rates through Kaplan–Meier analyses are rare. Hence in this study, we aim to evaluate the prognostic value of No. 10 lymphadenectomy for gastric cancer patients with total gastrectomy by survival analyses. The estimated overall 5-year survival rates were 46.44% and 37.43% in 10D+ group and 10D− group respectively, without significant difference (*P* = 0.3288). Goto et al^21^ reported that the index of estimated benefits from LN dissection of splenic hilar LNs was zero in patients who underwent a total gastrectomy with D2 LN dissection for Siewert type II AEG. Yamashita et al^22^ also found that the index of estimated benefit from No. 10 LN dissection was 0.7 for Siewert type II AEG patients. However, Kosuga et al^23^ demonstrated that the overall index of estimated benefits from LN dissection at the splenic hilum in patients who underwent a total gastrectomy curatively with simultaneous splenectomy was 5.49. Although our results showed that the 5-year survival rates between the 2 groups were not statistically significant, approximately 10% of 5-year survival rates in 10D− group yielded an inferior result as compared with that of 10D+ group. Moreover, the differences would have reached significances with using bigger patient samples, because type II error probably occurs on our results. Besides, the mean number of harvested LNs was higher in 10D+ group with significance as compared with that in 10D− group. Therefore, we presumed that the No. 10 LNs’ resection should be performed in total gastrectomy with D2 lymphadenectomy. Nevertheless, new studies using more patients are needed before stronger statement can be done. According to the results stratified by clinicopathologic factors, higher priority of No. 10 lymphadenectomies should be considered for patients with ages >60 years, Siewert II/ III tumors, N1 status, or IIIa/IIIc stages. However, for more advanced stage of gastric cancer, such as R1/R2 resection, T4b, N3b, M1, or IV stage, No. 10 lymphadenectomy is not necessary and should be avoided.

With respect to the safety, the fragile texture of the spleen and large amount of vessel branches being located at the splenic hilum may increase the risk of No. 10 LN resection. However, our results failed to show that there was significant difference in morbidity or mortality between 10D+ and 10D− group. Some spleen-preserved lymphadenectomy-related complications, such as intraperitoneal hemorrhage, or pancreatic leakage, had not occurred in the 2 groups. Only 1 patient from 10D+ group experienced intraoperative splenic injury. Studies have also reported that either laparoscopic spleen-preserving splenic hilum LNs’ dissection or open spleen-preserving No. 10 LNs’ resection could be performed safely.^24–26^ At the same time, with respect to the operation-related variables, there were no significant differences in terms of intraoperative blood loss volume, operation time, length of hospital stay, and reoperation rate between the 2 groups. So, the spleen-preserved No. 10 LN resection could be performed safely in clinic.

As in any other retrospective studies, limitation of the current analysis includes possible selection bias, detection bias, and performance of analysis bias.^27^ The possibility of inaccurate classification of the difference between the No. 11d and No. 10 remains a mystery. So, well-designed randomized controlled trials are needed to explore the effectiveness and safety of No. 10 LNs’ resection. Another limitation of this study is that the dissected No. 10 LNs of 106 patients were found to be fatty tissues by histological examination; in fact it can also be said that the surgeon and/or pathologist may have missed the positive nodes in these patients due to the presence of the fatty tissues which may have formed a veil. So, the results of metastatic ratio and metastatic degree may be biased due to this factor. However, the results of the survival analysis cannot be compromised. Furthermore, although major complications were not missed, some less severe complications could have been ignored.

## CONCLUSIONS

In conclusion, although the differences were obvious, the 5-year survival rates between the 2 groups did not reach statistical significances, which was probably caused by too small patient samples. High-quality studies with larger sample sizes are needed before stronger statement can be done. Until then, the No. 10 LNs’ resection might be recommended in total gastrectomy with D2 lymphadenectomy with an acceptable incidence of complications.
